# Supplementary material for: Causal Relationship between Aging and Anorexia Nervosa: A White-Matter-Microstructure-Mediated Mendelian Randomization Analysis
Source: Biomedicines. 2024 Aug 16;12(8):1874. doi: 10.3390/biomedicines12081874 (PMC11351342; doi:10.3390/biomedicines12081874)
Supplement: Supplementary file 1 [file biomedicines-12-01874-s001.zip › Supplementary Figure/Figure S8/Leaveoneout plot of MR analysis for TL to ubm-b-2011.pdf]

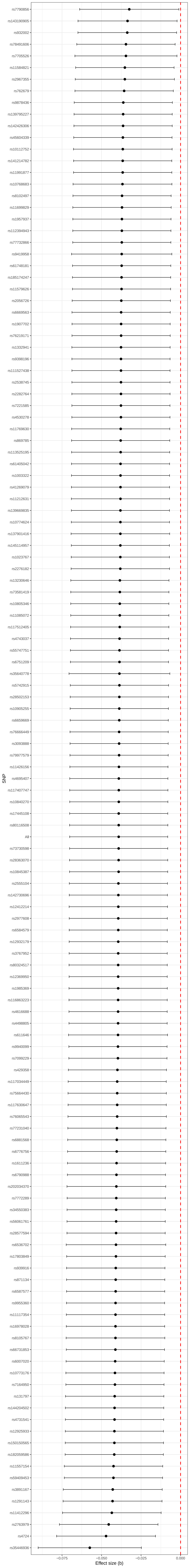
rs7790856
rs143190905
rs932002
rs78491606
rs7705526
rs11584821
rs2967355
rs762679
rs9878436
rs139795227
rs142426306
rs45604339
rs10112752
rs141214782
rs11991877
rs10768683
rs8102497
rs11699829
rs1957937
rs112394943
rs77732866
rs9419958
rs61748181
rs185174247
rs11579626
rs2056726
rs6669563
rs1907702
rs76219171
rs1332941
rs9398196
rs111527438
rs2538745
rs2282764
rs7221585
rs4530278
rs11769630
rs869785
rs113525195
rs61405042
rs1003322
rs41269079
rs11212631
rs139669835
rs10774624
rs137901416
rs145114957
rs1023767
rs2276182
rs13230646
rs73581419
rs10805346
rs11085072
rs117512405
rs4743037
rs55747751
rs6751209
rs35640778
rs5742915
rs28502153
rs10905255
rs6659669
rs76666449
rs3093888
rs79977579
rs11426156
rs4695407
rs117407747
rs10840270
rs17445108
rs80116508
All
rs73730598
rs28363070
rs10845387
rs2555104
rs142730696
rs12412214
rs2977608
rs6584579
rs12932179
rs3767952
rs80324517
rs12369950
rs1985369
rs116863223
rs4616688
rs4498805
rs611646
rs9940099
rs7099229
rs429358
rs117034449
rs75664430
rs117630647
rs76065543
rs77231040
rs6881568
rs6776756
rs1611236
rs6790988
rs202034370
rs7772289
rs34550383
rs56061761
rs28577594
rs6536702
rs17803849
rs939916
rs871134
rs6587577
rs9955360
rs11117354
rs16978028
rs8105767
rs66731853
rs6007020
rs10773176
rs7164950
rs131797
rs144204502
rs4731541
rs12925933
rs150150565
rs182059586
rs11557154
rs59409453
rs3891167
rs1291143
rs11412296
rs2763979
rs4724
rs35446936
SNP
−0.075
−0.050
−0.025
0.000
Effect size (b)
